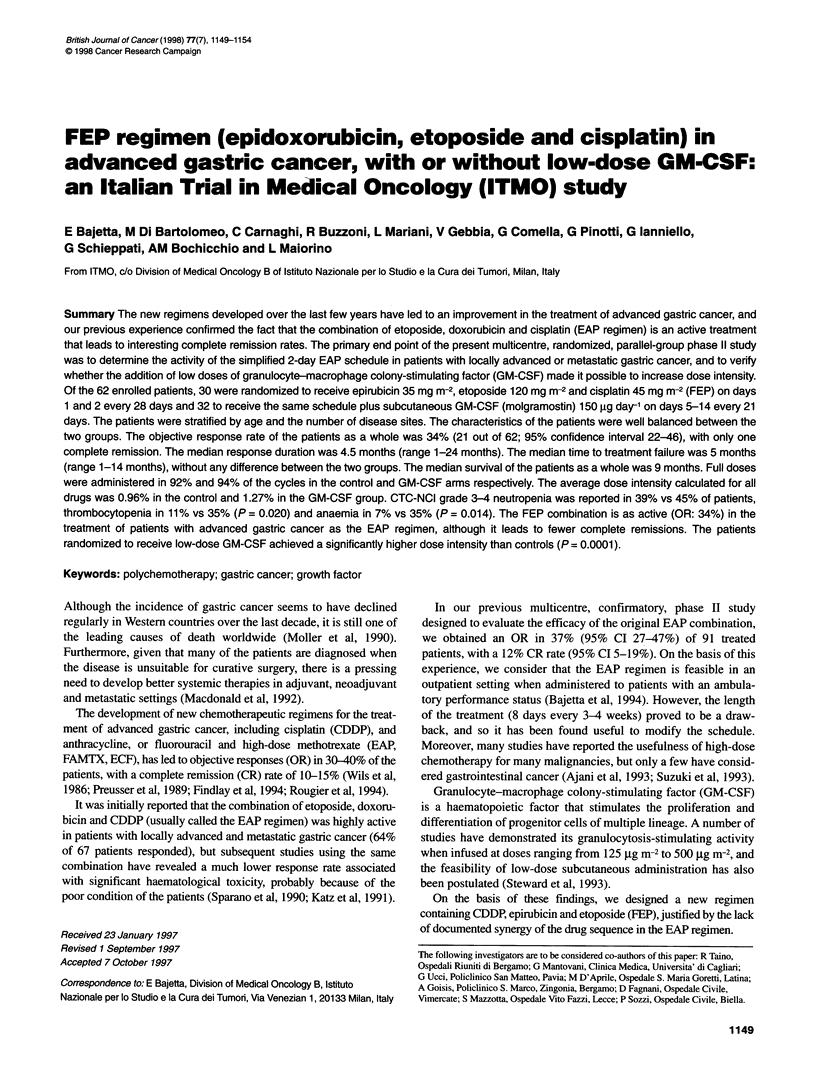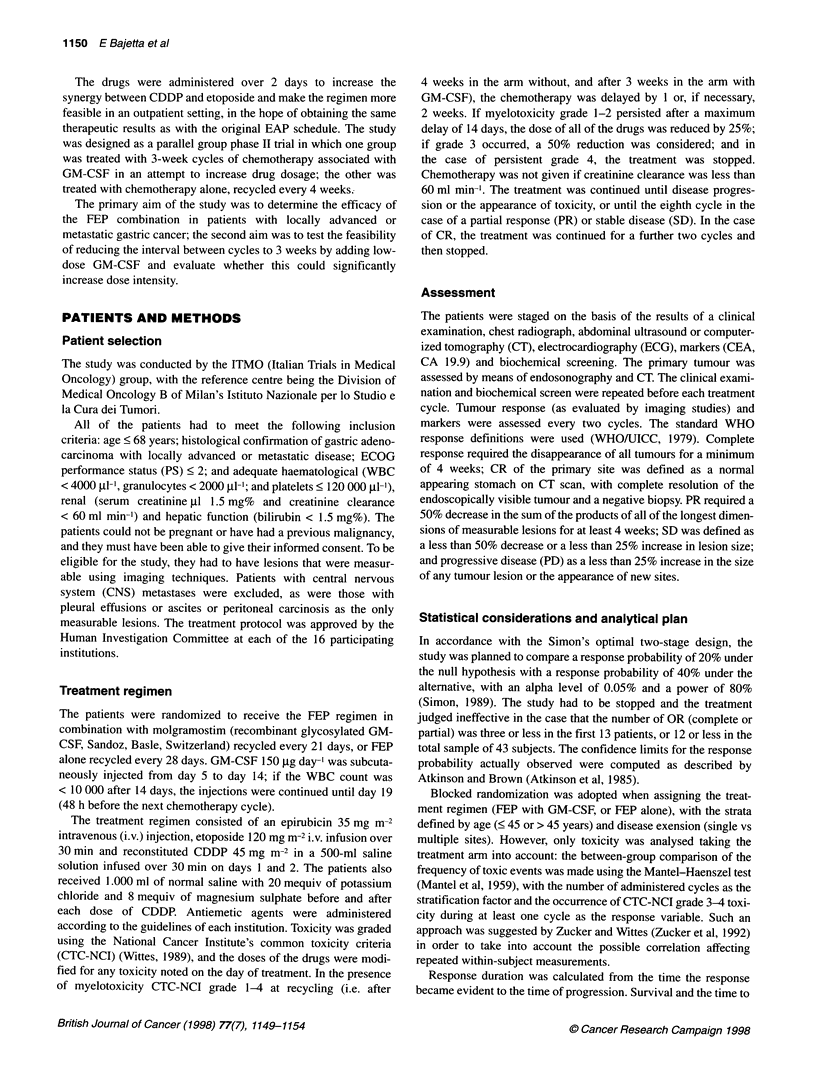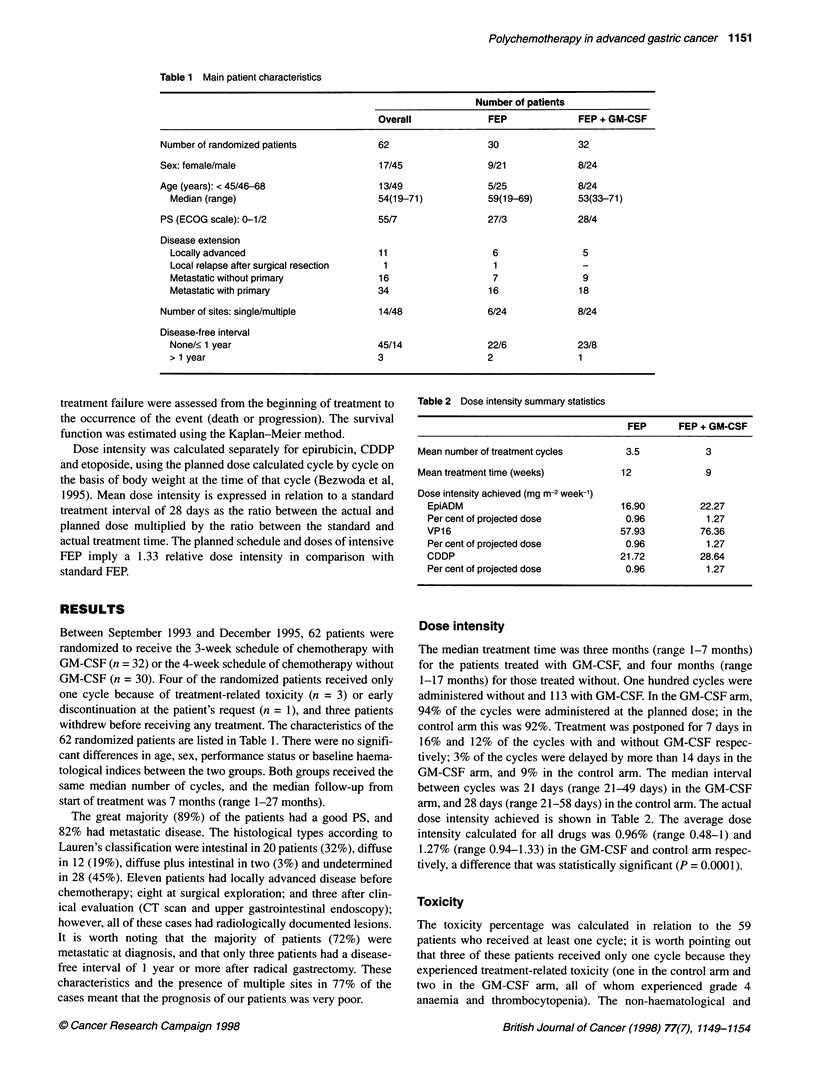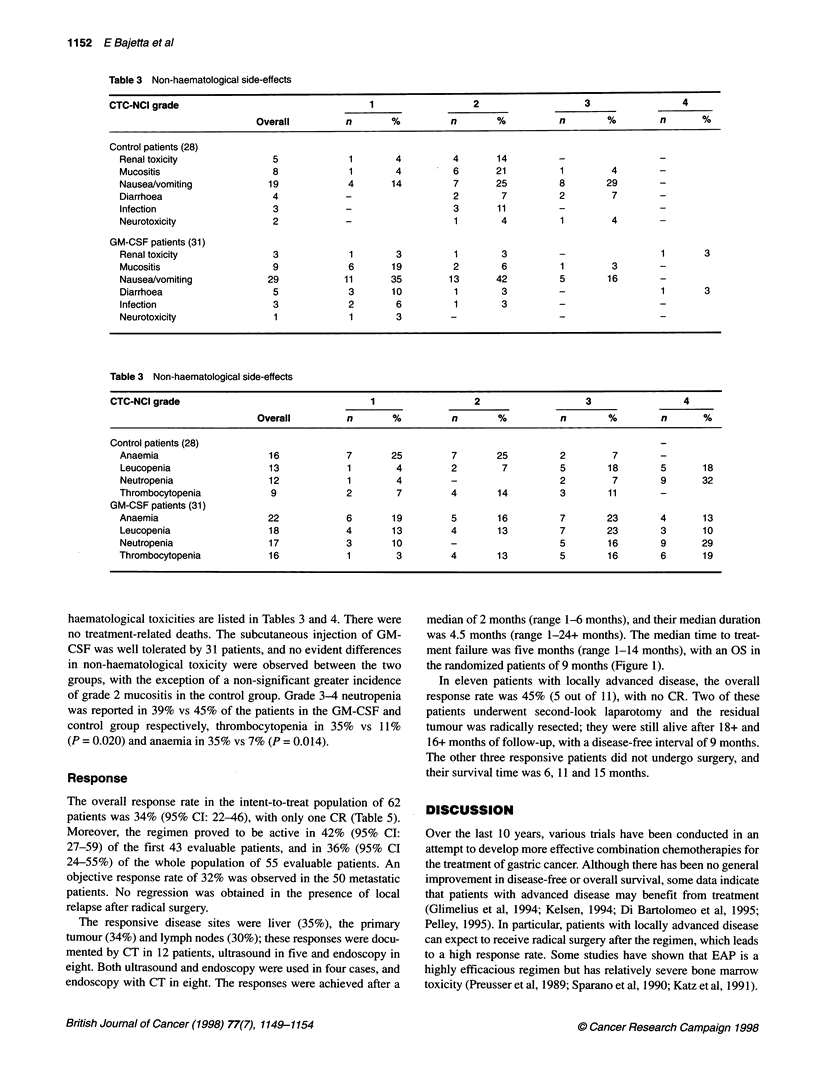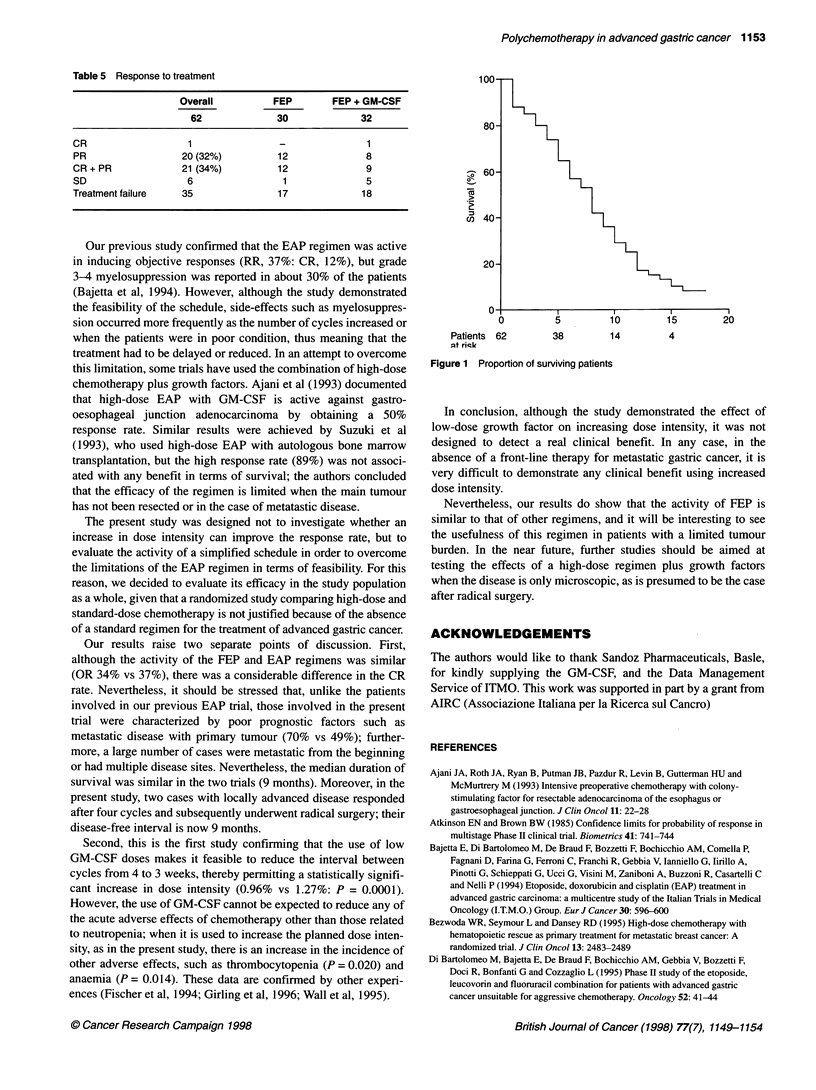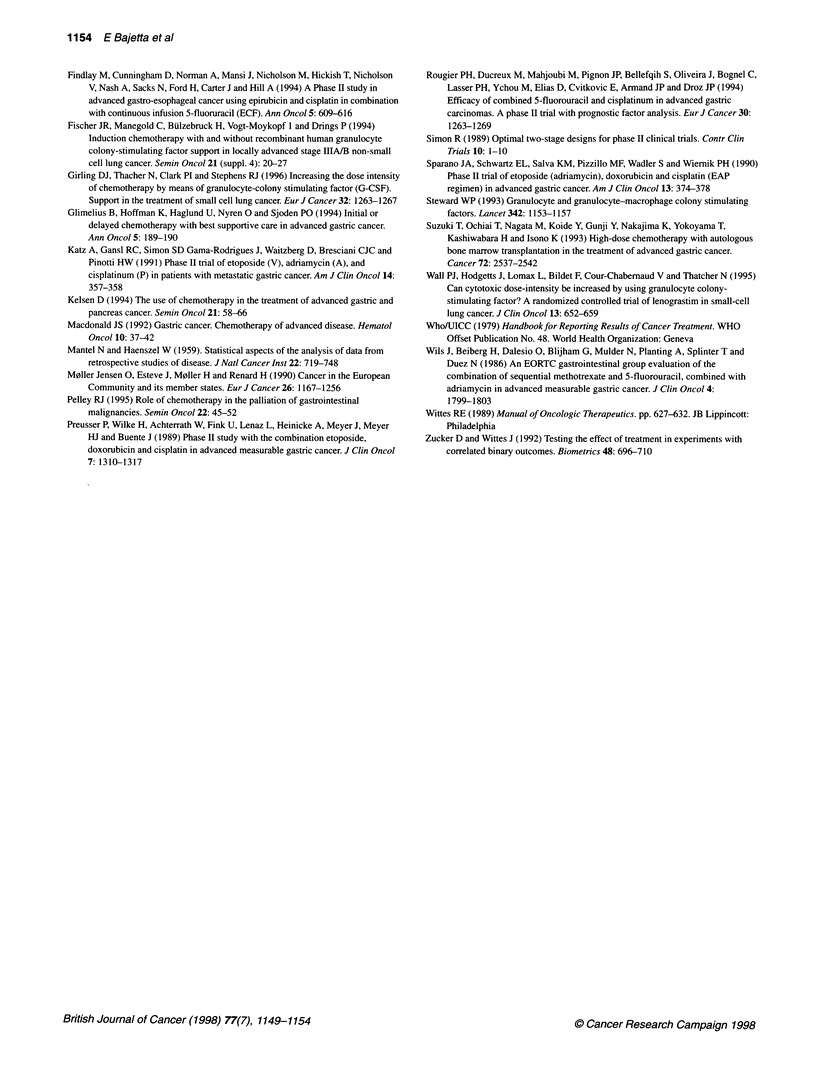# FEP regimen (epidoxorubicin, etoposide and cisplatin) in advanced gastric cancer, with or without low-dose GM-CSF: an Italian Trial in Medical Oncology (ITMO) study.

**DOI:** 10.1038/bjc.1998.191

**Published:** 1998-04

**Authors:** E. Bajetta, M. Di Bartolomeo, C. Carnaghi, R. Buzzoni, L. Mariani, V. Gebbia, G. Comella, G. Pinotti, G. Ianniello, G. Schieppati, A. M. Bochicchio, L. Maiorino

**Affiliations:** ITMO, c/o Division of Medical Oncology B of Istituto Nazionale per lo Studio e la Cura dei Tumori, Milan, Italy.

## Abstract

The new regimens developed over the last few years have led to an improvement in the treatment of advanced gastric cancer, and our previous experience confirmed the fact that the combination of etoposide, doxorubicin and cisplatin (EAP regimen) is an active treatment that leads to interesting complete remission rates. The primary end point of the present multicentre, randomized, parallel-group phase II study was to determine the activity of the simplified 2-day EAP schedule in patients with locally advanced or metastatic gastric cancer, and to verify whether the addition of low doses of granulocyte-macrophage colony-stimulating factor (GM-CSF) made it possible to increase dose intensity. Of the 62 enrolled patients, 30 were randomized to receive epirubicin 35 mg m(-2), etoposide 120 mg m(-2) and cisplatin 45 mg m(-2) (FEP) on days 1 and 2 every 28 days and 32 to receive the same schedule plus subcutaneous GM-CSF (molgramostin) 150 microg day(-1) on days 5-14 every 21 days. The patients were stratified by age and the number of disease sites. The characteristics of the patients were well balanced between the two groups. The objective response rate of the patients as a whole was 34% (21 out of 62; 95% confidence interval 22-46), with only one complete remission. The median response duration was 4.5 months (range 1-24 months). The median time to treatment failure was 5 months (range 1-14 months), without any difference between the two groups. The median survival of the patients as a whole was 9 months. Full doses were administered in 92% and 94% of the cycles in the control and GM-CSF arms respectively. The average dose intensity calculated for all drugs was 0.96% in the control and 1.27% in the GM-CSF group. CTC-NCI grade 3-4 neutropenia was reported in 39% vs 45% of patients, thrombocytopenia in 11% vs 35% (P = 0.020) and anaemia in 7% vs 35% (P = 0.014). The FEP combination is as active (OR: 34%) in the treatment of patients with advanced gastric cancer as the EAP regimen, although it leads to fewer complete remissions. The patients randomized to receive low-dose GM-CSF achieved a significantly higher dose intensity than controls (P = 0.0001).


British Journal of Cancer (1998) 77(7), 1149–1154
© 1998 Cancer Research Campaign

### FEP regimen (epidoxorubicin, etoposide and cisplatin) in advanced gastric cancer, with or without low-dose GM-CSF: an Italian Trial in Medical Oncology (ITMO) study

**E Bajetta, M Di Bartolomeo, C Carnaghi, R Buzzoni, L Mariani, V Gebbia, G Comella, G Pinotti, G Ianniello, G Schieppati, AM Bochicchio and L Maiorino**

From ITMO, c/o Division of Medical Oncology B of Istituto Nazionale per lo Studio e la Cura dei Tumori, Milan, Italy

**Summary** The new regimens developed over the last few years have led to an improvement in the treatment of advanced gastric cancer, and our previous experience confirmed the fact that the combination of etoposide, doxorubicin and cisplatin (EAP regimen) is an active treatment that leads to interesting complete remission rates. The primary end point of the present multicentre, randomized, parallel-group phase II study was to determine the activity of the simplified 2-day EAP schedule in patients with locally advanced or metastatic gastric cancer, and to verify whether the addition of low doses of granulocyte–macrophage colony-stimulating factor (GM-CSF) made it possible to increase dose intensity. Of the 62 enrolled patients, 30 were randomized to receive epirubicin 35 mg $m^{-2}$, etoposide 120 mg $m^{-2}$ and cisplatin 45 mg $m^{-2}$ (FEP) on days 1 and 2 every 28 days and 32 to receive the same schedule plus subcutaneous GM-CSF (molgramostin) 150 μg $day^{-1}$ on days 5–14 every 21 days. The patients were stratified by age and the number of disease sites. The characteristics of the patients were well balanced between the two groups. The objective response rate of the patients as a whole was 34% (21 out of 62; 95% confidence interval 22–46), with only one complete remission. The median response duration was 4.5 months (range 1–24 months). The median time to treatment failure was 5 months (range 1–14 months), without any difference between the two groups. The median survival of the patients as a whole was 9 months. Full doses were administered in 92% and 94% of the cycles in the control and GM-CSF arms respectively. The average dose intensity calculated for all drugs was 0.96% in the control and 1.27% in the GM-CSF group. CTC-NCI grade 3–4 neutropenia was reported in 39% vs 45% of patients, thrombocytopenia in 11% vs 35% ($P$ = 0.020) and anaemia in 7% vs 35% ($P$ = 0.014). The FEP combination is as active (OR: 34%) in the treatment of patients with advanced gastric cancer as the EAP regimen, although it leads to fewer complete remissions. The patients randomized to receive low-dose GM-CSF achieved a significantly higher dose intensity than controls ($P$ = 0.0001).

**Keywords:** polychemotherapy; gastric cancer; growth factor

Although the incidence of gastric cancer seems to have declined regularly in Western countries over the last decade, it is still one of the leading causes of death worldwide (Moller et al, 1990). Furthermore, given that many of the patients are diagnosed when the disease is unsuitable for curative surgery, there is a pressing need to develop better systemic therapies in adjuvant, neoadjuvant and metastatic settings (Macdonald et al, 1992).

The development of new chemotherapeutic regimens for the treatment of advanced gastric cancer, including cisplatin (CDDP), and anthracycline, or fluorouracil and high-dose methotrexate (EAP, FAMTX, ECF), has led to objective responses (OR) in 30–40% of the patients, with a complete remission (CR) rate of 10–15% (Wils et al, 1986; Preusser et al, 1989; Findlay et al, 1994; Rougier et al, 1994).

It was initially reported that the combination of etoposide, doxorubicin and CDDP (usually called the EAP regimen) was highly active in patients with locally advanced and metastatic gastric cancer (64% of 67 patients responded), but subsequent studies using the same combination have revealed a much lower response rate associated with significant haematological toxicity, probably because of the poor condition of the patients (Sparano et al, 1990; Katz et al, 1991).

*Received 23 January 1997*
*Revised 1 September 1997*
*Accepted 7 October 1997*

*Correspondence to:* E Bajetta, Division of Medical Oncology B, Istituto Nazionale per lo Studio e la Cura dei Tumori, Via Venezian 1, 20133 Milan, Italy

In our previous multicentre, confirmatory, phase II study designed to evaluate the efficacy of the original EAP combination, we obtained an OR in 37% (95% CI 27–47%) of 91 treated patients, with a 12% CR rate (95% CI 5–19%). On the basis of this experience, we consider that the EAP regimen is feasible in an outpatient setting when administered to patients with an ambulatory performance status (Bajetta et al, 1994). However, the length of the treatment (8 days every 3–4 weeks) proved to be a drawback, and so it has been found useful to modify the schedule. Moreover, many studies have reported the usefulness of high-dose chemotherapy for many malignancies, but only a few have considered gastrointestinal cancer (Ajani et al, 1993; Suzuki et al, 1993).

Granulocyte–macrophage colony-stimulating factor (GM-CSF) is a haematopoietic factor that stimulates the proliferation and differentiation of progenitor cells of multiple lineage. A number of studies have demonstrated its granulocytosis-stimulating activity when infused at doses ranging from 125 μg $m^{-2}$ to 500 μg $m^{-2}$, and the feasibility of low-dose subcutaneous administration has also been postulated (Steward et al, 1993).

On the basis of these findings, we designed a new regimen containing CDDP, epirubicin and etoposide (FEP), justified by the lack of documented synergy of the drug sequence in the EAP regimen.

The following investigators are to be considered co-authors of this paper: R Taino, Ospedali Riuniti di Bergamo; G Mantovani, Clinica Medica, Universita' di Cagliari; G Ucci, Policlinico San Matteo, Pavia; M D'Aprile, Ospedale S. Maria Goretti, Latina; A Goisis, Policlinico S. Marco, Zingonia, Bergamo; D Fagnani, Ospedale Civile, Vimercate; S Mazzotta, Ospedale Vito Fazzi, Lecce; P Sozzi, Ospedale Civile, Biella.

The drugs were administered over 2 days to increase the synergy between CDDP and etoposide and make the regimen more feasible in an outpatient setting, in the hope of obtaining the same therapeutic results as with the original EAP schedule. The study was designed as a parallel group phase II trial in which one group was treated with 3-week cycles of chemotherapy associated with GM-CSF in an attempt to increase drug dosage; the other was treated with chemotherapy alone, recycled every 4 weeks.

The primary aim of the study was to determine the efficacy of the FEP combination in patients with locally advanced or metastatic gastric cancer; the second aim was to test the feasibility of reducing the interval between cycles to 3 weeks by adding low-dose GM-CSF and evaluate whether this could significantly increase dose intensity.

### PATIENTS AND METHODS

### Patient selection

The study was conducted by the ITMO (Italian Trials in Medical Oncology) group, with the reference centre being the Division of Medical Oncology B of Milan's Istituto Nazionale per lo Studio e la Cura dei Tumori.

All of the patients had to meet the following inclusion criteria: age ≤ 68 years; histological confirmation of gastric adenocarcinoma with locally advanced or metastatic disease; ECOG performance status (PS) ≤ 2; and adequate haematological (WBC < 4000 μl$^{-1}$, granulocytes < 2000 μl$^{-1}$; and platelets ≤ 120 000 μl$^{-1}$), renal (serum creatinine μl 1.5 mg% and creatinine clearance < 60 ml min$^{-1}$) and hepatic function (bilirubin < 1.5 mg%). The patients could not be pregnant or have had a previous malignancy, and they must have been able to give their informed consent. To be eligible for the study, they had to have lesions that were measurable using imaging techniques. Patients with central nervous system (CNS) metastases were excluded, as were those with pleural effusions or ascites or peritoneal carcinosis as the only measurable lesions. The treatment protocol was approved by the Human Investigation Committee at each of the 16 participating institutions.

### Treatment regimen

The patients were randomized to receive the FEP regimen in combination with molgramostim (recombinant glycosylated GM-CSF, Sandoz, Basle, Switzerland) recycled every 21 days, or FEP alone recycled every 28 days. GM-CSF 150 μg day$^{-1}$ was subcutaneously injected from day 5 to day 14; if the WBC count was < 10 000 after 14 days, the injections were continued until day 19 (48 h before the next chemotherapy cycle).

The treatment regimen consisted of an epirubicin 35 mg m$^{-2}$ intravenous (i.v.) injection, etoposide 120 mg m$^{-2}$ i.v. infusion over 30 min and reconstituted CDDP 45 mg m$^{-2}$ in a 500-ml saline solution infused over 30 min on days 1 and 2. The patients also received 1.000 ml of normal saline with 20 mequiv of potassium chloride and 8 mequiv of magnesium sulphate before and after each dose of CDDP. Antiemetic agents were administered according to the guidelines of each institution. Toxicity was graded using the National Cancer Institute's common toxicity criteria (CTC-NCI) (Wittes, 1989), and the doses of the drugs were modified for any toxicity noted on the day of treatment. In the presence of myelotoxicity CTC-NCI grade 1–4 at recycling (i.e. after 4 weeks in the arm without, and after 3 weeks in the arm with GM-CSF), the chemotherapy was delayed by 1 or, if necessary, 2 weeks. If myelotoxicity grade 1–2 persisted after a maximum delay of 14 days, the dose of all of the drugs was reduced by 25%; if grade 3 occurred, a 50% reduction was considered; and in the case of persistent grade 4, the treatment was stopped. Chemotherapy was not given if creatinine clearance was less than 60 ml min$^{-1}$. The treatment was continued until disease progression or the appearance of toxicity, or until the eighth cycle in the case of a partial response (PR) or stable disease (SD). In the case of CR, the treatment was continued for a further two cycles and then stopped.

### Assessment

The patients were staged on the basis of the results of a clinical examination, chest radiograph, abdominal ultrasound or computerized tomography (CT), electrocardiography (ECG), markers (CEA, CA 19.9) and biochemical screening. The primary tumour was assessed by means of endosonography and CT. The clinical examination and biochemical screen were repeated before each treatment cycle. Tumour response (as evaluated by imaging studies) and markers were assessed every two cycles. The standard WHO response definitions were used (WHO/UICC, 1979). Complete response required the disappearance of all tumours for a minimum of 4 weeks; CR of the primary site was defined as a normal appearing stomach on CT scan, with complete resolution of the endoscopically visible tumour and a negative biopsy. PR required a 50% decrease in the sum of the products of all of the longest dimensions of measurable lesions for at least 4 weeks; SD was defined as a less than 50% decrease or a less than 25% increase in lesion size; and progressive disease (PD) as a less than 25% increase in the size of any tumour lesion or the appearance of new sites.

### Statistical considerations and analytical plan

In accordance with the Simon's optimal two-stage design, the study was planned to compare a response probability of 20% under the null hypothesis with a response probability of 40% under the alternative, with an alpha level of 0.05% and a power of 80% (Simon, 1989). The study had to be stopped and the treatment judged ineffective in the case that the number of OR (complete or partial) was three or less in the first 13 patients, or 12 or less in the total sample of 43 subjects. The confidence limits for the response probability actually observed were computed as described by Atkinson and Brown (Atkinson et al, 1985).

Blocked randomization was adopted when assigning the treatment regimen (FEP with GM-CSF, or FEP alone), with the strata defined by age (≤ 45 or > 45 years) and disease exension (single vs multiple sites). However, only toxicity was analysed taking the treatment arm into account: the between-group comparison of the frequency of toxic events was made using the Mantel–Haenszel test (Mantel et al, 1959), with the number of administered cycles as the stratification factor and the occurrence of CTC-NCI grade 3–4 toxicity during at least one cycle as the response variable. Such an approach was suggested by Zucker and Wittes (Zucker et al, 1992) in order to take into account the possible correlation affecting repeated within-subject measurements.

Response duration was calculated from the time the response became evident to the time of progression. Survival and the time to

© Cancer Research Campaign 1998

**Table 1** Main patient characteristics

| | Number of patients | | |
|---|---|---|---|
| | **Overall** | **FEP** | **FEP + GM-CSF** |
| Number of randomized patients | 62 | 30 | 32 |
| Sex: female/male | 17/45 | 9/21 | 8/24 |
| Age (years): < 45/46–68 | 13/49 | 5/25 | 8/24 |
| Median (range) | 54(19–71) | 59(19–69) | 53(33–71) |
| PS (ECOG scale): 0–1/2 | 55/7 | 27/3 | 28/4 |
| Disease extension | | | |
| Locally advanced | 11 | 6 | 5 |
| Local relapse after surgical resection | 1 | 1 | – |
| Metastatic without primary | 16 | 7 | 9 |
| Metastatic with primary | 34 | 16 | 18 |
| Number of sites: single/multiple | 14/48 | 6/24 | 8/24 |
| Disease-free interval | | | |
| None/≤ 1 year | 45/14 | 22/6 | 23/8 |
| > 1 year | 3 | 2 | 1 |

treatment failure were assessed from the beginning of treatment to the occurrence of the event (death or progression). The survival function was estimated using the Kaplan–Meier method.

Dose intensity was calculated separately for epirubicin, CDDP and etoposide, using the planned dose calculated cycle by cycle on the basis of body weight at the time of that cycle (Bezwoda et al, 1995). Mean dose intensity is expressed in relation to a standard treatment interval of 28 days as the ratio between the actual and planned dose multiplied by the ratio between the standard and actual treatment time. The planned schedule and doses of intensive FEP imply a 1.33 relative dose intensity in comparison with standard FEP.

### RESULTS

Between September 1993 and December 1995, 62 patients were randomized to receive the 3-week schedule of chemotherapy with GM-CSF ($n = 32$) or the 4-week schedule of chemotherapy without GM-CSF ($n = 30$). Four of the randomized patients received only one cycle because of treatment-related toxicity ($n = 3$) or early discontinuation at the patient's request ($n = 1$), and three patients withdrew before receiving any treatment. The characteristics of the 62 randomized patients are listed in Table 1. There were no significant differences in age, sex, performance status or baseline haematological indices between the two groups. Both groups received the same median number of cycles, and the median follow-up from start of treatment was 7 months (range 1–27 months).

The great majority (89%) of the patients had a good PS, and 82% had metastatic disease. The histological types according to Lauren's classification were intestinal in 20 patients (32%), diffuse in 12 (19%), diffuse plus intestinal in two (3%) and undetermined in 28 (45%). Eleven patients had locally advanced disease before chemotherapy; eight at surgical exploration; and three after clinical evaluation (CT scan and upper gastrointestinal endoscopy); however, all of these cases had radiologically documented lesions. It is worth noting that the majority of patients (72%) were metastatic at diagnosis, and that only three patients had a disease-free interval of 1 year or more after radical gastrectomy. These characteristics and the presence of multiple sites in 77% of the cases meant that the prognosis of our patients was very poor.

**Table 2** Dose intensity summary statistics

| | **FEP** | **FEP + GM-CSF** |
|---|---|---|
| Mean number of treatment cycles | 3.5 | 3 |
| Mean treatment time (weeks) | 12 | 9 |
| Dose intensity achieved (mg m$^{-2}$ week$^{-1}$) | | |
| EpiADM | 16.90 | 22.27 |
| Per cent of projected dose | 0.96 | 1.27 |
| VP16 | 57.93 | 76.36 |
| Per cent of projected dose | 0.96 | 1.27 |
| CDDP | 21.72 | 28.64 |
| Per cent of projected dose | 0.96 | 1.27 |

### Dose intensity

The median treatment time was three months (range 1–7 months) for the patients treated with GM-CSF, and four months (range 1–17 months) for those treated without. One hundred cycles were administered without and 113 with GM-CSF. In the GM-CSF arm, 94% of the cycles were administered at the planned dose; in the control arm this was 92%. Treatment was postponed for 7 days in 16% and 12% of the cycles with and without GM-CSF respectively; 3% of the cycles were delayed by more than 14 days in the GM-CSF arm, and 9% in the control arm. The median interval between cycles was 21 days (range 21–49 days) in the GM-CSF arm, and 28 days (range 21–58 days) in the control arm. The actual dose intensity achieved is shown in Table 2. The average dose intensity calculated for all drugs was 0.96% (range 0.48–1) and 1.27% (range 0.94–1.33) in the GM-CSF and control arm respectively, a difference that was statistically significant ($P = 0.0001$).

### Toxicity

The toxicity percentage was calculated in relation to the 59 patients who received at least one cycle; it is worth pointing out that three of these patients received only one cycle because they experienced treatment-related toxicity (one in the control arm and two in the GM-CSF arm, all of whom experienced grade 4 anaemia and thrombocytopenia). The non-haematological and

© Cancer Research Campaign 1998

**Table 3** Non-haematological side-effects

| CTC-NCI grade | | 1 | | 2 | | 3 | | 4 | |
|---|---|---|---|---|---|---|---|---|---|
| | Overall | *n* | % | *n* | % | *n* | % | *n* | % |
| Control patients (28) | | | | | | | | | |
| Renal toxicity | 5 | 1 | 4 | 4 | 14 | – | | – | |
| Mucositis | 8 | 1 | 4 | 6 | 21 | 1 | 4 | – | |
| Nausea/vomiting | 19 | 4 | 14 | 7 | 25 | 8 | 29 | – | |
| Diarrhoea | 4 | – | | 2 | 7 | 2 | 7 | – | |
| Infection | 3 | – | | 3 | 11 | – | | – | |
| Neurotoxicity | 2 | – | | 1 | 4 | 1 | 4 | – | |
| GM-CSF patients (31) | | | | | | | | | |
| Renal toxicity | 3 | 1 | 3 | 1 | 3 | – | | 1 | 3 |
| Mucositis | 9 | 6 | 19 | 2 | 6 | 1 | 3 | – | |
| Nausea/vomiting | 29 | 11 | 35 | 13 | 42 | 5 | 16 | – | |
| Diarrhoea | 5 | 3 | 10 | 1 | 3 | – | | 1 | 3 |
| Infection | 3 | 2 | 6 | 1 | 3 | – | | – | |
| Neurotoxicity | 1 | 1 | 3 | – | | – | | – | |

**Table 3** Non-haematological side-effects

| CTC-NCI grade | | 1 | | 2 | | 3 | | 4 | |
|---|---|---|---|---|---|---|---|---|---|
| | Overall | *n* | % | *n* | % | *n* | % | *n* | % |
| Control patients (28) | | | | | | | | – | |
| Anaemia | 16 | 7 | 25 | 7 | 25 | 2 | 7 | – | |
| Leucopenia | 13 | 1 | 4 | 2 | 7 | 5 | 18 | 5 | 18 |
| Neutropenia | 12 | 1 | 4 | – | | 2 | 7 | 9 | 32 |
| Thrombocytopenia | 9 | 2 | 7 | 4 | 14 | 3 | 11 | – | |
| GM-CSF patients (31) | | | | | | | | | |
| Anaemia | 22 | 6 | 19 | 5 | 16 | 7 | 23 | 4 | 13 |
| Leucopenia | 18 | 4 | 13 | 4 | 13 | 7 | 23 | 3 | 10 |
| Neutropenia | 17 | 3 | 10 | – | | 5 | 16 | 9 | 29 |
| Thrombocytopenia | 16 | 1 | 3 | 4 | 13 | 5 | 16 | 6 | 19 |

haematological toxicities are listed in Tables 3 and 4. There were no treatment-related deaths. The subcutaneous injection of GM-CSF was well tolerated by 31 patients, and no evident differences in non-haematological toxicity were observed between the two groups, with the exception of a non-significant greater incidence of grade 2 mucositis in the control group. Grade 3–4 neutropenia was reported in 39% vs 45% of the patients in the GM-CSF and control group respectively, thrombocytopenia in 35% vs 11% ($P = 0.020$) and anaemia in 35% vs 7% ($P = 0.014$).

### Response

The overall response rate in the intent-to-treat population of 62 patients was 34% (95% CI: 22–46), with only one CR (Table 5). Moreover, the regimen proved to be active in 42% (95% CI: 27–59) of the first 43 evaluable patients, and in 36% (95% CI 24–55%) of the whole population of 55 evaluable patients. An objective response rate of 32% was observed in the 50 metastatic patients. No regression was obtained in the presence of local relapse after radical surgery.

The responsive disease sites were liver (35%), the primary tumour (34%) and lymph nodes (30%); these responses were documented by CT in 12 patients, ultrasound in five and endoscopy in eight. Both ultrasound and endoscopy were used in four cases, and endoscopy with CT in eight. The responses were achieved after a median of 2 months (range 1–6 months), and their median duration was 4.5 months (range 1–24+ months). The median time to treatment failure was five months (range 1–14 months), with an OS in the randomized patients of 9 months (Figure 1).

In eleven patients with locally advanced disease, the overall response rate was 45% (5 out of 11), with no CR. Two of these patients underwent second-look laparotomy and the residual tumour was radically resected; they were still alive after 18+ and 16+ months of follow-up, with a disease-free interval of 9 months. The other three responsive patients did not undergo surgery, and their survival time was 6, 11 and 15 months.

### DISCUSSION

Over the last 10 years, various trials have been conducted in an attempt to develop more effective combination chemotherapies for the treatment of gastric cancer. Although there has been no general improvement in disease-free or overall survival, some data indicate that patients with advanced disease may benefit from treatment (Glimelius et al, 1994; Kelsen, 1994; Di Bartolomeo et al, 1995; Pelley, 1995). In particular, patients with locally advanced disease can expect to receive radical surgery after the regimen, which leads to a high response rate. Some studies have shown that EAP is a highly efficacious regimen but has relatively severe bone marrow toxicity (Preusser et al, 1989; Sparano et al, 1990; Katz et al, 1991).

© Cancer Research Campaign 1998

**Table 5** Response to treatment

| | Overall 62 | FEP 30 | FEP + GM-CSF 32 |
|---|---|---|---|
| CR | 1 | – | 1 |
| PR | 20 (32%) | 12 | 8 |
| CR + PR | 21 (34%) | 12 | 9 |
| SD | 6 | 1 | 5 |
| Treatment failure | 35 | 17 | 18 |

Our previous study confirmed that the EAP regimen was active in inducing objective responses (RR, 37%: CR, 12%), but grade 3–4 myelosuppression was reported in about 30% of the patients (Bajetta et al, 1994). However, although the study demonstrated the feasibility of the schedule, side-effects such as myelosuppression occurred more frequently as the number of cycles increased or when the patients were in poor condition, thus meaning that the treatment had to be delayed or reduced. In an attempt to overcome this limitation, some trials have used the combination of high-dose chemotherapy plus growth factors. Ajani et al (1993) documented that high-dose EAP with GM-CSF is active against gastro-oesophageal junction adenocarcinoma by obtaining a 50% response rate. Similar results were achieved by Suzuki et al (1993), who used high-dose EAP with autologous bone marrow transplantation, but the high response rate (89%) was not associated with any benefit in terms of survival; the authors concluded that the efficacy of the regimen is limited when the main tumour has not been resected or in the case of metatastic disease.

The present study was designed not to investigate whether an increase in dose intensity can improve the response rate, but to evaluate the activity of a simplified schedule in order to overcome the limitations of the EAP regimen in terms of feasibility. For this reason, we decided to evaluate its efficacy in the study population as a whole, given that a randomized study comparing high-dose and standard-dose chemotherapy is not justified because of the absence of a standard regimen for the treatment of advanced gastric cancer.

Our results raise two separate points of discussion. First, although the activity of the FEP and EAP regimens was similar (OR 34% vs 37%), there was a considerable difference in the CR rate. Nevertheless, it should be stressed that, unlike the patients involved in our previous EAP trial, those involved in the present trial were characterized by poor prognostic factors such as metastatic disease with primary tumour (70% vs 49%); furthermore, a large number of cases were metastatic from the beginning or had multiple disease sites. Nevertheless, the median duration of survival was similar in the two trials (9 months). Moreover, in the present study, two cases with locally advanced disease responded after four cycles and subsequently underwent radical surgery; their disease-free interval is now 9 months.

Second, this is the first study confirming that the use of low GM-CSF doses makes it feasible to reduce the interval between cycles from 4 to 3 weeks, thereby permitting a statistically significant increase in dose intensity (0.96% vs 1.27%: $P$ = 0.0001). However, the use of GM-CSF cannot be expected to reduce any of the acute adverse effects of chemotherapy other than those related to neutropenia; when it is used to increase the planned dose intensity, as in the present study, there is an increase in the incidence of other adverse effects, such as thrombocytopenia ($P$ = 0.020) and anaemia ($P$ = 0.014). These data are confirmed by other experiences (Fischer et al, 1994; Girling et al, 1996; Wall et al, 1995).

**Figure 1** Proportion of surviving patients

In conclusion, although the study demonstrated the effect of low-dose growth factor on increasing dose intensity, it was not designed to detect a real clinical benefit. In any case, in the absence of a front-line therapy for metastatic gastric cancer, it is very difficult to demonstrate any clinical benefit using increased dose intensity.

Nevertheless, our results do show that the activity of FEP is similar to that of other regimens, and it will be interesting to see the usefulness of this regimen in patients with a limited tumour burden. In the near future, further studies should be aimed at testing the effects of a high-dose regimen plus growth factors when the disease is only microscopic, as is presumed to be the case after radical surgery.

### ACKNOWLEDGEMENTS

The authors would like to thank Sandoz Pharmaceuticals, Basle, for kindly supplying the GM-CSF, and the Data Management Service of ITMO. This work was supported in part by a grant from AIRC (Associazione Italiana per la Ricerca sul Cancro)

### REFERENCES

Ajani JA, Roth JA, Ryan B, Putman JB, Pazdur R, Levin B, Gutterman HU and McMurtrery M (1993) Intensive preoperative chemotherapy with colony-stimulating factor for resectable adenocarcinoma of the esophagus or gastroesophageal junction. *J Clin Oncol* **11**: 22–28

Atkinson EN and Brown BW (1985) Confidence limits for probability of response in multistage Phase II clinical trial. *Biometrics* **41**: 741–744

Bajetta E, Di Bartolomeo M, De Braud F, Bozzetti F, Bochicchio AM, Comella P, Fagnani D, Farina G, Ferroni C, Franchi R, Gebbia V, Ianniello G, Iirillo A, Pinotti G, Schieppati G, Ucci G, Visini M, Zaniboni A, Buzzoni R, Casartelli C and Nelli P (1994) Etoposide, doxorubicin and cisplatin (EAP) treatment in advanced gastric carcinoma: a multicentre study of the Italian Trials in Medical Oncology (I.T.M.O.) Group. *Eur J Cancer* **30**: 596–600

Bezwoda WR, Seymour L and Dansey RD (1995) High-dose chemotherapy with hematopoietic rescue as primary treatment for metastatic breast cancer: A randomized trial. *J Clin Oncol* **13**: 2483–2489

Di Bartolomeo M, Bajetta E, De Braud F, Bochicchio AM, Gebbia V, Bozzetti F, Doci R, Bonfanti G and Cozzaglio L (1995) Phase II study of the etoposide, leucovorin and fluoruracil combination for patients with advanced gastric cancer unsuitable for aggressive chemotherapy. *Oncology* **52**: 41–44

© Cancer Research Campaign 1998

Findlay M, Cunningham D, Norman A, Mansi J, Nicholson M, Hickish T, Nicholson V, Nash A, Sacks N, Ford H, Carter J and Hill A (1994) A Phase II study in advanced gastro-esophageal cancer using epirubicin and cisplatin in combination with continuous infusion 5-fluoruracil (ECF). *Ann Oncol* **5**: 609–616

Fischer JR, Manegold C, Bülzebruck H, Vogt-Moykopf 1 and Drings P (1994) Induction chemotherapy with and without recombinant human granulocyte colony-stimulating factor support in locally advanced stage IIIA/B non-small cell lung cancer. *Semin Oncol* **21** (suppl. 4): 20–27

Girling DJ, Thacher N, Clark PI and Stephens RJ (1996) Increasing the dose intensity of chemotherapy by means of granulocyte-colony stimulating factor (G-CSF). Support in the treatment of small cell lung cancer. *Eur J Cancer* **32**: 1263–1267

Glimelius B, Hoffman K, Haglund U, Nyren O and Sjoden PO (1994) Initial or delayed chemotherapy with best supportive care in advanced gastric cancer. *Ann Oncol* **5**: 189–190

Katz A, Gansl RC, Simon SD Gama-Rodrigues J, Waitzberg D, Bresciani CJC and Pinotti HW (1991) Phase II trial of etoposide (V), adriamycin (A), and cisplatinum (P) in patients with metastatic gastric cancer. *Am J Clin Oncol* **14**: 357–358

Kelsen D (1994) The use of chemotherapy in the treatment of advanced gastric and pancreas cancer. *Semin Oncol* **21**: 58–66

Macdonald JS (1992) Gastric cancer. Chemotherapy of advanced disease. *Hematol Oncol* **10**: 37–42

Mantel N and Haenszel W (1959). Statistical aspects of the analysis of data from retrospective studies of disease. *J Natl Cancer Inst* **22**: 719–748

Møller Jensen O, Esteve J, Møller H and Renard H (1990) Cancer in the European Community and its member states. *Eur J Cancer* **26**: 1167–1256

Pelley RJ (1995) Role of chemotherapy in the palliation of gastrointestinal malignancies. *Semin Oncol* **22**: 45–52

Preusser P, Wilke H, Achterrath W, Fink U, Lenaz L, Heinicke A, Meyer J, Meyer HJ and Buente J (1989) Phase II study with the combination etoposide, doxorubicin and cisplatin in advanced measurable gastric cancer. *J Clin Oncol* **7**: 1310–1317

Rougier PH, Ducreux M, Mahjoubi M, Pignon JP, Bellefqih S, Oliveira J, Bognel C, Lasser PH, Ychou M, Elias D, Cvitkovic E, Armand JP and Droz JP (1994) Efficacy of combined 5-fluorouracil and cisplatinum in advanced gastric carcinomas. A phase II trial with prognostic factor analysis. *Eur J Cancer* **30**: 1263–1269

Simon R (1989) Optimal two-stage designs for phase II clinical trials. *Contr Clin Trials* **10**: 1–10

Sparano JA, Schwartz EL, Salva KM, Pizzillo MF, Wadler S and Wiernik PH (1990) Phase II trial of etoposide (adriamycin), doxorubicin and cisplatin (EAP regimen) in advanced gastric cancer. *Am J Clin Oncol* **13**: 374–378

Steward WP (1993) Granulocyte and granulocyte–macrophage colony stimulating factors. *Lancet* **342**: 1153–1157

Suzuki T, Ochiai T, Nagata M, Koide Y, Gunji Y, Nakajima K, Yokoyama T, Kashiwabara H and Isono K (1993) High-dose chemotherapy with autologous bone marrow transplantation in the treatment of advanced gastric cancer. *Cancer* **72**: 2537–2542

Wall PJ, Hodgetts J, Lomax L, Bildet F, Cour-Chabernaud V and Thatcher N (1995) Can cytotoxic dose-intensity be increased by using granulocyte colony-stimulating factor? A randomized controlled trial of lenograstim in small-cell lung cancer. *J Clin Oncol* **13**: 652–659

Who/UICC (1979) *Handbook for Reporting Results of Cancer Treatment*. WHO Offset Publication No. 48. World Health Organization: Geneva

Wils J, Beiberg H, Dalesio O, Blijham G, Mulder N, Planting A, Splinter T and Duez N (1986) An EORTC gastrointestinal group evaluation of the combination of sequential methotrexate and 5-fluorouracil, combined with adriamycin in advanced measurable gastric cancer. *J Clin Oncol* **4**: 1799–1803

Wittes RE (1989) *Manual of Oncologic Therapeutics*. pp. 627–632. JB Lippincott: Philadelphia

Zucker D and Wittes J (1992) Testing the effect of treatment in experiments with correlated binary outcomes. *Biometrics* **48**: 696–710

*© Cancer Research Campaign 1998*